# Cyanotic and Unresponsive: A Diagnostic Challenge in Alkyl Nitrite-Induced Methaemoglobinaemia

**DOI:** 10.7759/cureus.98627

**Published:** 2025-12-07

**Authors:** Sayantan Basu, Rayna Koshy, Verghese Kurien, Joseph Allen, Purba Chakrabarty

**Affiliations:** 1 General Medicine, Eastbourne District General Hospital, Eastbourne, GBR; 2 Internal Medicine, New Cross Hospital, Royal Wolverhampton NHS Trust, Wolverhampton, GBR; 3 General Internal Medicine, East Sussex Healthcare NHS Trust, Eastbourne, GBR; 4 Internal Medicine, Eastbourne District General Hospital, Eastbourne, GBR

**Keywords:** alkyl nitrites (poppers), diagnostic challenge, g6pd deficiency, hypoxia, jehovah’s witness, methemoglobinemia, methylene blue, vulnerable adult

## Abstract

Methaemoglobinaemia is a potentially serious condition in which haemoglobin is oxidised to a form that cannot carry oxygen effectively. Recreational inhalation of alkyl nitrites (“poppers”) may precipitate this condition and can be overlooked when the presentation is sudden and severe.

We report a 53-year-old male of Chinese ethnicity, with multiple comorbidities, including learning disabilities, who collapsed in public and presented unresponsive, cyanotic, hypothermic, and profoundly hypoxic despite high‑flow oxygen. Arterial blood gas analysis revealed a high methaemoglobin level with a normal arterial oxygen tension, producing a marked saturation gap. A bottle of alkyl nitrites found in his possession supported a toxic cause. Intravenous methylene blue led to rapid clinical and biochemical improvement, and the patient made a full recovery. Safeguarding measures were implemented due to his cognitive vulnerability.

This case highlights the diagnostic challenge of unexplained hypoxia, the essential role of co‑oximetry in confirming methaemoglobinaemia, considerations for enzyme deficiencies like G6PD, and the importance of harm-reduction strategies amid rising poppers use.

## Introduction

Methaemoglobinaemia occurs when haemoglobin is oxidised to the ferric (Fe³⁺) state, preventing effective oxygen binding and delivery [1]. Severe cases may result in tissue hypoxia, neurological depression, and cardiovascular instability. It can be congenital (e.g., due to cytochrome b5 reductase deficiency, more common in populations like Native Americans or Ashkenazi Jews) or acquired, often from toxins or drugs.

Recreational alkyl nitrites (“poppers”) are a common acquired cause inducing methaemoglobinaemia through nitrite-mediated oxidation, though presentations may be delayed or unrecognised [2,3].

Diagnosis is particularly challenging when patients present unresponsive or without collateral history. The characteristic “saturation gap”, i.e., low pulse oximetry readings despite normal arterial oxygen tension, should prompt consideration of methaemoglobinaemia or other dyshaemoglobinaemias. Co‑oximetry, performed as part of arterial blood gas analysis, is critical for diagnosis because it directly quantifies methaemoglobin levels, unlike conventional pulse oximetry, which becomes unreliable at high methaemoglobin concentrations and typically plateaus near 85% [4].

Epidemiologically, recreational alkyl nitrite use has been rising in the UK, with national surveys consistently showing higher prevalence among younger adults and particularly within men who have sex with men (MSM) communities. Data from the Crime Survey for England and Wales indicate that poppers remain one of the most commonly used non-controlled psychoactive substances among young adults, and Public Health England reports have similarly highlighted high lifetime use within MSM populations. These trends underscore the need for clinician awareness and targeted public health interventions [2,3].

## Case presentation

A 53-year-old male of Chinese ethnicity was found collapsed outside a shop after vomiting. On arrival of paramedics, he was unresponsive with a Glasgow Coma Scale score of 3, central and peripheral cyanosis, profoundly hypothermic, and oxygen saturation of 60% while receiving 15 L/min oxygen via a non-rebreather mask. His radial pulse was weak, and no reliable history was available.

The patient had a medical background of learning disability, atrial fibrillation treated with a direct oral anticoagulant, epilepsy, and a previous cerebrovascular accident. He was also a Jehovah’s Witness and declined blood products. His regular medications were rivaroxaban, bisoprolol, and levetiracetam.

Imaging

**Figure 1:**
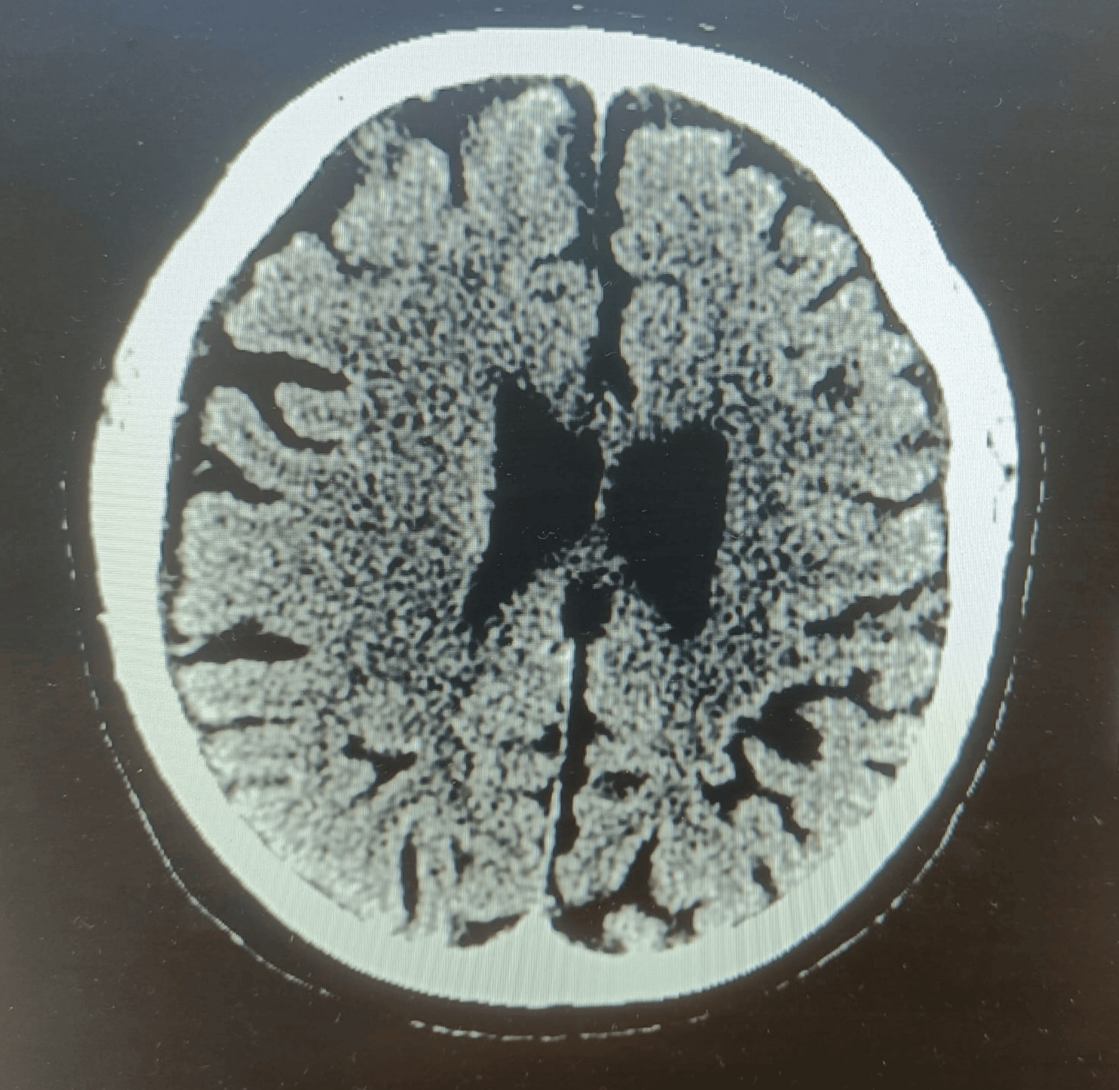
Non-contrast CT of the head demonstrates no acute intracranial abnormality.

A non-contrast CT of the head was performed on arrival, revealing no acute intracranial abnormality (Figure 1).

Arterial blood gas and laboratory findings

Arterial blood gas analysis taken while the patient was receiving high inspired oxygen revealed features consistent with methaemoglobinaemia. Co-oximetry demonstrated a methaemoglobin concentration > 30%, with a normal arterial oxygen tension and reduced clinical oxygen saturation, confirming a significant saturation gap.

Routine laboratory results were otherwise unremarkable. Laboratory findings are summarised in Table 1.

**Table 1:** Arterial blood gas analysis showing methaemoglobinaemia and routine laboratory blood test results. ABG: arterial blood gas; pCO2: partial pressure of carbon dioxide; pO2: partial pressure of oxygen; sO2: oxygen saturation; ALT: alanine aminotransferase; CRP: C-reactive protein; WCC: white cell count; RBC: red blood cells.

Test	Result	Units	Reference range
pH	7.37	-	7.35–7.45
pCO2	5.1	kPa	4.7–6.0
pO2	63.5	kPa	10.5–13.5 (on room air)
Bicarbonate	20.8	mmol/L	22–28
Lactate	1.0	mmol/L	0.5–2.2
Methaemoglobin	>30	%	<1.5%
sO2 (ABG-derived)	93.0	%	>95%
Sodium	140	mmol/L	133-146
Potassium	4.4	mmol/L	3.5-5.3
Urea	6.0	mmol/L	2.5-7.8
Creatinine	56	µmol/L	59-104
Bilirubin	11	µmol/L	0-21
Alkaline phosphatase	54	U/L	30-130
ALT	20	U/L	10-50
CRP	1	mg/L	0-5
Corrected calcium	2.19	mmol/L	2.20-2.60
Haemoglobin	144	g/L	130-180
WCC	10.81	×10⁹/L	4.0-11
Neutrophils	9.79	×10⁹/L	2-7.5
RBC	4.69	×1012/L	4.5-6.5

The identification of a bottle of alkyl nitrites in the patient’s pocket raised suspicion for toxin‑induced methaemoglobinaemia.

Management

Toxicology guidance recommended immediate administration of intravenous methylthioninium chloride (methylene blue). He was given 150 mg intravenously (based on an estimated weight of 75 kg). A repeat arterial blood gas at 30 minutes showed a reduction in methaemoglobin to 12%, and a second half-dose was administered. Serial arterial blood gases with co-oximetry were performed in the intensive care unit at 30-minute intervals until normalisation. Active warming with a forced-air device was used to treat hypothermia. Blood-based therapies were avoided in accordance with the patient’s religious beliefs. Methaemoglobin levels normalised to 1.1%. Due to concerns regarding cognitive vulnerability and lack of collateral information, the patient was referred to adult social care for a safeguarding assessment.

Outcome

He made a full neurological and physiological recovery and returned to his baseline level of function. He was transferred from intensive care to the ward and discharged with follow-up, including harm-reduction counselling and safeguarding support.

## Discussion

This case underscores the diagnostic challenge of methaemoglobinaemia, particularly in an unresponsive patient without collateral history, where the presentation can easily mimic seizure, stroke, or toxic ingestion. In this instance, several comorbidities, including epilepsy, atrial fibrillation, and a previous cerebrovascular accident, further broadened the initial differential diagnosis and obscured the underlying cause.

A key diagnostic clue was the presence of a saturation gap: severe hypoxia on pulse oximetry despite a high arterial partial pressure of oxygen (PaO₂). This discrepancy should prompt consideration of a dyshaemoglobinaemia. Understanding the limitations of pulse oximetry is essential. Pulse oximetry relies only on two wavelengths and assumes the presence of oxyhaemoglobin and deoxyhaemoglobin alone; it becomes unreliable when abnormal haemoglobin species such as methaemoglobin are present. Co-oximetry, by contrast, uses multiple wavelengths to directly quantify individual haemoglobin species, including methaemoglobin, carboxyhaemoglobin, and sulfhaemoglobin, making it indispensable for confirming dyshaemoglobinaemia and accurately interpreting the saturation gap.

Intravenous methylthioninium chloride (methylene blue) at 1-2 mg/kg remains the recommended first-line treatment for methaemoglobinaemia, as it accelerates the nicotinamide adenine dinucleotide phosphate (NADPH)-dependent reduction of methaemoglobin back to functional haemoglobin. However, methylene blue can be ineffective or precipitate oxidative haemolysis in individuals with glucose-6-phosphate dehydrogenase (G6PD) deficiency, an X-linked condition more common in people of East or Southeast Asian, Mediterranean, and African ancestry, with several variants such as the Canton and Kaiping types frequently reported in Chinese populations. Methylene blue requires NADPH generated via the G6PD pathway to exert its effect, and hence, patients with significant deficiency may fail to respond or may clinically deteriorate. In these patients, alternatives would include ascorbic acid, exchange transfusion or hyperbaric oxygen as required. Although G6PD testing was not performed in our patient, since delaying antidotal therapy would have been unsafe, the patient’s rapid clinical and biochemical improvement without evidence of haemolysis strongly suggests that meaningful G6PD deficiency was unlikely. Awareness of this association nonetheless remains essential when treating patients from higher-prevalence ethnic groups [5].

Recreational use of alkyl nitrites (“poppers”) is increasing, particularly among men who have sex with men (for their euphoric and muscle-relaxant effects during receptive anal intercourse), club-goers, and younger adults. Although legally sold as “room odourisers” or “leather cleaners”, their recreational inhalation is often mistakenly viewed as harmless [6]. Severe methaemoglobinaemia is likely under-reported due to stigma, reluctance to disclose use, and limited clinician familiarity [2,3]. This is concerning, as most affected individuals are otherwise healthy young people who can make a full and rapid recovery if the condition is recognised promptly and methylene blue is administered without delay.

The rising incidence of nitrite-induced methaemoglobinaemia highlights the need for earlier recognition and improved diagnostic capability. In our case, access to a pre-hospital arterial blood gas analyser with integrated co-oximetry could have established the diagnosis at the scene, enabling immediate toxicology input and advance preparation of methylene blue prior to hospital arrival, potentially reducing the duration of profound tissue hypoxia. However, routine UK ambulance services do not carry arterial blood gas analysers or co-oximetry devices because of cost, calibration demands, and operational constraints, making early confirmation of methaemoglobinaemia impossible in most pre-hospital settings.

Encouragingly, emerging 2025 data, including pilot pre-hospital studies presented at the European Respiratory Society (ERS) and American Thoracic Society (ATS) meetings, demonstrate that portable multi-wavelength co-oximeters can reliably detect dyshaemoglobinaemias in the field. As these devices become more robust, affordable, and user-friendly, integrating point-of-care co-oximetry into frontline paramedic practice is increasingly feasible. Although national guidelines have not yet incorporated this technology, its wider adoption offers the potential for faster, on-scene diagnosis and reduced delays in administering definitive therapy.

Given that methaemoglobinaemia is both entirely reversible and profoundly time-sensitive, earlier recognition is crucial. This reinforces the need for clinician vigilance, non-judgmental history-taking, and advocacy for emerging diagnostic tools alongside targeted harm-reduction strategies [7].

## Conclusions

Methaemoglobinaemia should be considered in any patient with sudden cyanosis and hypoxia that does not improve with oxygen therapy, especially when a saturation gap is present. Co-oximetry is essential for confirming the diagnosis, and early administration of methylene blue usually results in rapid reversal. Non-judgmental history-taking and awareness of inhaled nitrite exposure are important when assessing unexplained hypoxia. Earlier recognition in the pre-hospital and emergency settings, supported by improved access to point-of-care testing, has the potential to reduce avoidable morbidity. Prompt identification transforms a potentially life-threatening presentation into a readily reversible condition.
